# Supplementary material for: Comparative multiomics analysis of cell physiological state after culture in a basket bioreactor
Source: Sci Rep. 2022 Nov 23;12:20161. doi: 10.1038/s41598-022-24687-4 (PMC9686226; doi:10.1038/s41598-022-24687-4)

54712554:+@NW_023666033.1:54715083:54715191:+@NW_023666033.1:54715847:54715963:+@NW_02366603

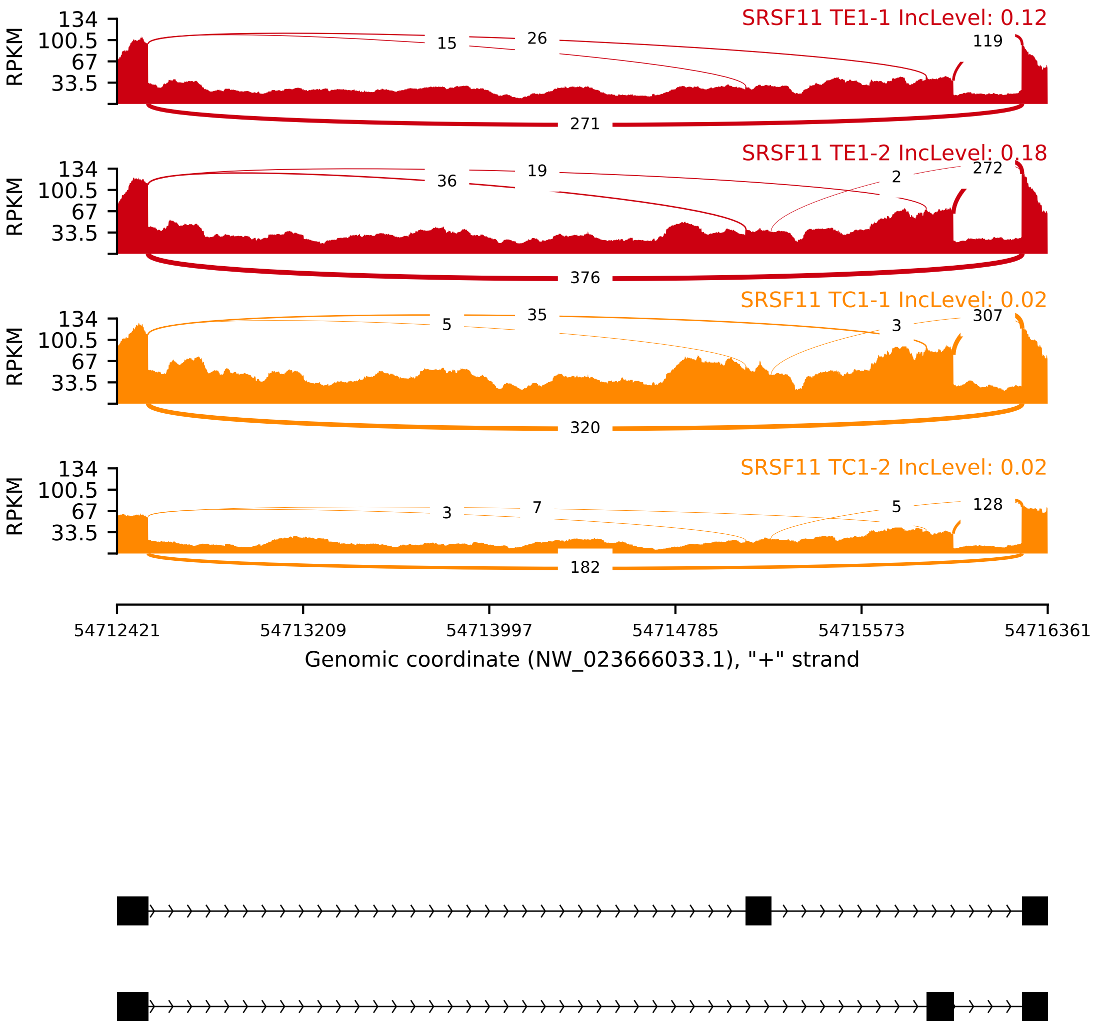

Supplement: Supplementary file 1 — Supplementary Information 1. [file 41598_2022_24687_MOESM1_ESM.zip › raw data/Transcriptomic raw data/7.AS/2.dasplot/TE1vsTC1/MXE/15_SRSF11_NW_023666033.1_54712421_54712554_+@NW_023666033.1_54715083_54715191_+@NW_023666033.1_54715847_54715963_+@NW_023666033.1_54716253_54716362_+.pdf]
